# Effects of Practicing With and Obtaining Crowdsourced Feedback From the Video-Based Communication Assessment App on Resident Physicians’ Adverse Event Communication Skills: Pre-post Trial

**DOI:** 10.2196/40758

**Published:** 2022-10-03

**Authors:** Andrew A White, Ann M King, Angelo E D’Addario, Karen Berg Brigham, Suzanne Dintzis, Emily E Fay, Thomas H Gallagher, Kathleen M Mazor

**Affiliations:** 1 Department of Medicine University of Washington School of Medicine Seattle, WA United States; 2 National Board of Medical Examiners Philadelphia, PA United States; 3 Collaborative for Accountability and Improvement University of Washington Seattle, WA United States; 4 Department of Pathology University of Washington School of Medicine Seattle, WA United States; 5 Department of Obstetrics and Gynecology University of Washington School of Medicine Seattle, WA United States; 6 Meyers Primary Care Institute University of Massachusetts Medical School Worcester, MA United States

**Keywords:** medical error disclosure, simulation studies, communication assessment, graduate medical education, crowdsourcing, patient-centered care, medical education, virtual education, virtual communication, physician communication, resident, virtual learning, digital learning, video communication, medical error, digital response

## Abstract

**Background:**

US residents require practice and feedback to meet Accreditation Council for Graduate Medical Education mandates and patient expectations for effective communication after harmful errors. Current instructional approaches rely heavily on lectures, rarely provide individualized feedback to residents about communication skills, and may not assure that residents acquire the skills desired by patients. The Video-based Communication Assessment (VCA) app is a novel tool for simulating communication scenarios for practice and obtaining crowdsourced assessments and feedback on physicians’ communication skills. We previously established that crowdsourced laypeople can reliably assess residents’ error disclosure skills with the VCA app. However, its efficacy for error disclosure training has not been tested.

**Objective:**

We aimed to evaluate the efficacy of using VCA practice and feedback as a stand-alone intervention for the development of residents’ error disclosure skills.

**Methods:**

We conducted a pre-post study in 2020 with pathology, obstetrics and gynecology, and internal medicine residents at an academic medical center in the United States. At baseline, residents each completed 2 specialty-specific VCA cases depicting medical errors. Audio responses were rated by at least 8 crowdsourced laypeople using 6 items on a 5-point scale. At 4 weeks, residents received numerical and written feedback derived from layperson ratings and then completed 2 additional cases. Residents were randomly assigned cases at baseline and after feedback assessments to avoid ordinal effects. Ratings were aggregated to create overall assessment scores for each resident at baseline and after feedback. Residents completed a survey of demographic characteristics. We used a 2×3 split-plot ANOVA to test the effects of time (pre-post) and specialty on communication ratings.

**Results:**

In total, 48 residents completed 2 cases at time 1, received a feedback report at 4 weeks, and completed 2 more cases. The mean ratings of residents’ communication were higher at time 2 versus time 1 (3.75 vs 3.53; *P*<.001). Residents with prior error disclosure experience performed better at time 1 compared to those without such experience (ratings: mean 3.63 vs mean 3.46; *P*=.02). No differences in communication ratings based on specialty or years in training were detected. Residents’ communication was rated higher for angry cases versus sad cases (mean 3.69 vs mean 3.58; *P*=.01). Less than half of all residents (27/62, 44%) reported prior experience with disclosing medical harm to patients; experience differed significantly among specialties (*P*<.001) and was lowest for pathology (1/17, 6%).

**Conclusions:**

Residents at all training levels can potentially improve error disclosure skills with VCA practice and feedback. Error disclosure curricula should prepare residents for responding to various patient affects. Simulated error disclosure may particularly benefit trainees in diagnostic specialties, such as pathology, with infrequent real-life error disclosure practice opportunities. Future research should examine the effectiveness, feasibility, and acceptability of VCA within a longitudinal error disclosure curriculum.

## Introduction

Communication after medical harm typically falls short of patients’ and families’ needs [1]. Patients often feel abandoned, confused, and uncertain about how to obtain information and support [2]. Because many physicians are unprepared for these conversations [3,4], in 2017, the US Accreditation Council for Graduate Medical Education (ACGME) required all residents to receive training on how to disclose adverse events and participate in the “real or simulated” disclosure of harm events to patients and families [5]. Unfortunately, many US residency programs do not assure that graduates achieve competency in these skills. In a 2021 survey of over 11,000 US residents, 28% of respondents reported receiving no training in error disclosure. Of those who did, only 9.2% underwent simulation training [6]. Instead, most received lecture-based training or informal training—techniques with critical limitations for developing communication skills that require practice and feedback.

Although some error disclosure curricula may improve knowledge and attitudes, published interventions lack rigorous assessments of skill acquisition [7]. Residents can learn through clinical practice, but high-stakes disclosures are infrequent, are problematic to observe, and are seldom accompanied by formative feedback from supervisors or harmed patients [8,9]. Lectures and multiple-choice questions do not assure skill acquisition [7], and simulations with standardized patients are logistically demanding, are expensive to implement at scale, and are lacking in statistical reliability [10-14]. It is also unknown whether standardized patients and faculty physician raters adequately approximate the viewpoints of patients who have experienced medical injuries. In particular, physicians’ viewpoints about ideal disclosure content and performance differ from those of patients, potentially limiting faculties’ ability to assess and provide coaching on performance [9,15]. To fully meet ACGME requirements and patient expectations, educators need tools for residents to practice error disclosure and receive formative feedback that is patient-centered, reliable, prompt, affordable, and practical.

The US National Board of Medical Examiners created the Video-based Communication Assessment (VCA) app to allow physicians and trainees to practice and receive specific individual feedback on their verbal communication [16,17]. The VCA app presents brief videos of case vignettes and prompts users to audio-record what they would say to the patient. Laypeople recruited on Amazon’s Mechanical Turk (MTurk) rate the responses as if they were the patient in the vignette [18]. MTurk has a very large, diverse population for crowdsourcing, along with extensive proof for being an inexpensive, rapid, and high-quality data source [19-21]. VCA users receive feedback reports with ratings, comparative data on their cohorts, learning points derived from raters’ comments, and sample audio recordings of highly rated peer responses. In a study of primary care communication scenarios, the VCA app provided high-quality, actionable feedback [22]. We adapted this tool for harmful medical error cases and found that crowdsourced laypeople provide reliable assessments of physician error disclosure and that adequately sized panels of crowdsourced laypeople can serve as reliable surrogates for panels of patients with past involvement in harmful errors [23]. However, the effects of VCA practice and feedback on residents’ error disclosure skills are unknown.

This paper describes a pre-post trial of using VCA practice and feedback to improve residents’ error disclosure skills. This approach involved using the VCA app as the vehicle for both training and assessment as residents sequentially practiced communication skills, received the results of the assessments and feedback to guide improvement, and then responded again to assess skill growth. We hypothesized that residents’ error disclosure skills, as rated by laypeople, would improve with personalized feedback. We also sought to evaluate 2 secondary research questions. First, we hypothesized that residents’ performance would vary with patient affect. Second, based on prior literature [24,25], we hypothesized that residents’ confidence with error disclosure would not correlate with laypersons’ ratings of their communication skills.

## Methods

### Overview

**Figure 1:**
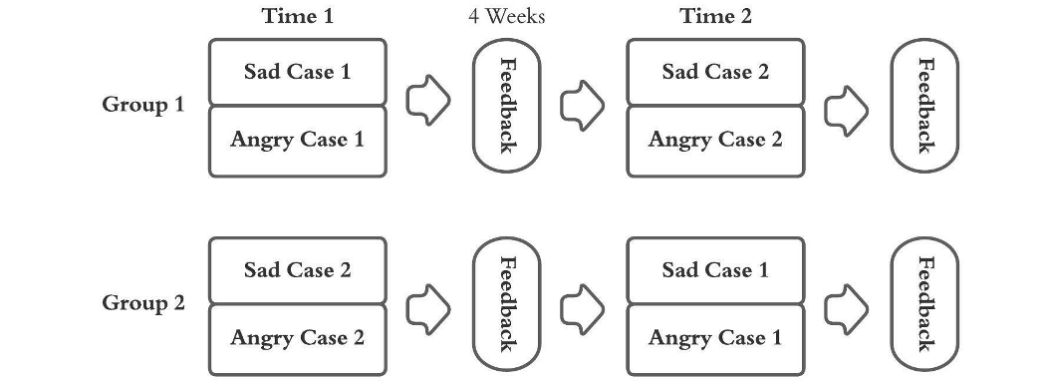
The crossover study design for 48 residents who used the Video-based Communication Assessment app to practice error disclosure and receive feedback.

This pre-post efficacy study of the VCA app for error disclosure was conducted to determine whether laypersons’ ratings of resident error disclosure improve with practice and feedback. With input from experienced attending physicians, we made 12 cases, which included 4 cases specific to the following three fields: internal medicine, pathology, and obstetrics and gynecology (OBGYN). Each case consisted of 3 or 4 vignettes depicting sequential stages in a conversation, such as initially sharing information about a mistake or responding to a patient’s emotional response (Multimedia Appendix 1). We recruited resident physicians at an academic center to complete each of the four cases (ie, 2 cases at 2 time points). Participating residents were randomly assigned to 1 of 2 groups within their specialty that differed only by the order of case assignment (Figure 1). Residents completed 2 cases at time 1; feedback was provided via the app after approximately 4 weeks, and residents were asked to complete the remaining two cases at time 2. The time points were spaced by 4 weeks, so that residents could be recruited while they were available during teaching conferences. We asked residents to not discuss the cases with colleagues until after study completion. We collected crowdsourced ratings of residents’ performances and surveys from residents.

### VCA Software and Content

The VCA app was described previously [16,23], and this project used the same software for presenting vignettes, recording residents’ responses, and delivering feedback. For each specialty, 2 cases portrayed an angry patient response, and another 2 cases portrayed a sad emotional response. Professional actors portrayed the patients. We designed cases to be of equivalent error severity. Further, 2 attending physicians and 3 senior residents pilot-tested the cases in their specialty and provided feedback via structured interviews to improve the cases’ relevance, quality, and believability.

**Figure 2:**
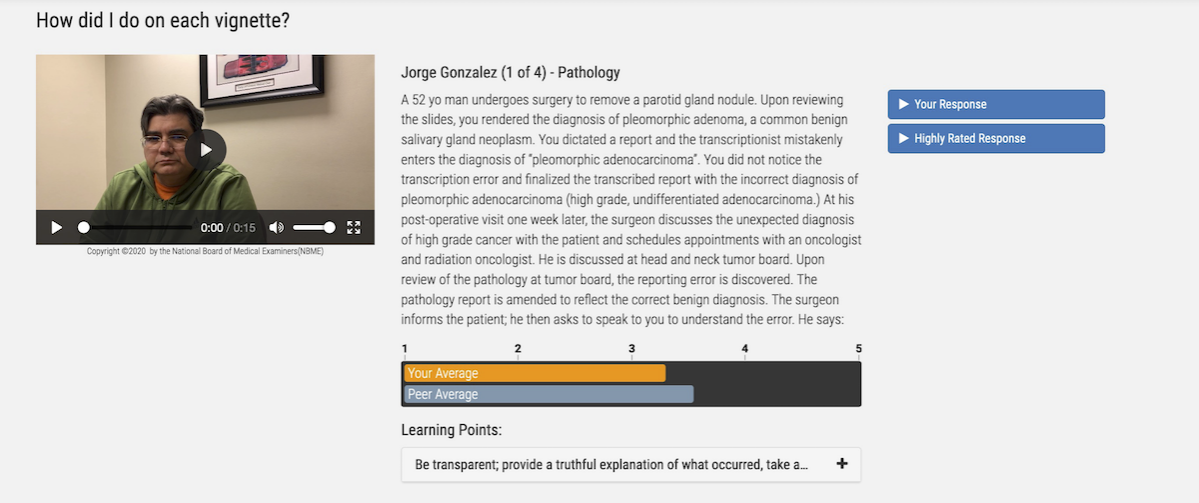
A screenshot from the Video-based Communication Assessment app displaying feedback for a single vignette within a case and the user controls for replaying the vignette video, replaying the user's response, replaying a highly rated response, and expanding the field containing advice regarding what laypeople wish the physician would say.

Feedback reports, which were viewed in the VCA app, presented users with their overall communication scores for each vignette, the average scores of their peers, and written advice derived from the comments of raters who responded to the following question: “what would you want the provider to say if you were the patient in this situation?” Users could replay their own responses and listen to a highly rated response from a peer (Figure 2).

### Setting and Participants

We recruited residents from March to June 2020 at all training levels (postgraduate years 1-4) from 3 departments at the University of Washington. The departments represented a procedural specialty (OBGYN), a diagnostic specialty (pathology), and a nonprocedural specialty (internal medicine). In the United States, pathology and OBGYN residency training typically lasts 4 years, and internal medicine residency training lasts 3 years. We invited all 237 OBGYN (n=28), internal medicine (n=183), and pathology (n=26) residents. Recruitment for the pathology and OBGYN specialties occurred at program-wide teaching conferences, with protected time for VCA use. The COVID-19 pandemic disrupted the in-person internal medicine conferences that were planned for recruitment; most internal medicine residents were recruited via email invitation. Each conference included a 10-minute orientation to VCA and 30 minutes for completing 2 cases. Residents received a US $50 gift card after completing all 4 VCA cases. At the time, the residencies did not have required program-wide training for error disclosure, and this trial did not provide didactic training.

### Ethical Considerations

The University of Washington Institutional Review Board determined that this study was exempt from a review of resident, layperson, and patient advocate participants based on its policies, procedures, and guidance (case identifier: STUDY00008246) [26]. Risks and benefits were explained verbally; consent was implied by voluntary participation in the VCA exercise.

### Outcomes Measured

Residents provided audio responses to each vignette through the VCA software. Audio responses were bundled into rating tasks for MTurk layperson raters who met the following criteria: US residents, those aged 18 years or older, and those able to speak and read English. Raters for OBGYN vignettes were further restricted to women. To participate, raters completed an audio check, answered demographic questions, read a description of the vignette that was written in lay language, viewed the patient video, and listened to the first resident response. The raters then responded to 6 items that covered domains related to accountability, honesty, apology, empathy, caring, and overall response (Multimedia Appendix 2) before advancing to the next response. The items used a 5-point scale that was labeled with “Poor,” “Fair,” “Good,” “Very good,” and “Excellent.” The instrument was developed by the investigators because the core competency assessment tool developed by the US ACGME only measures residents’ disclosure of patient safety events as “participates,” “discloses,” or “models disclosure,” rather than assessing disclosure quality or the patient experience of disclosure [27]. Although a very limited number of tools exist for rating residents’ error disclosure to standardized patients [7], they include questions about body language or are intended for faculty raters; therefore, they were not appropriate for incorporation in the VCA app. After rating the last response in the set, the raters were prompted to enter free text in response to the following question: “What would you want the provider to say if you were the patient in this situation?” Crowdsourced raters received variable compensation amounts based on a rate of US $0.20 per rating.

Residents were surveyed before and after each video-based communication assessment with the questionnaires that were built into the VCA app. The initial survey asked about the residents’ sex, their level of training, and whether they had personally disclosed medical harm to a patient. Residents answered the following two items, using a 5-point scale ranging from “strongly agree” to “strongly disagree”: “I am confident in my ability to share information with a patient after medical harm” and “I am confident in my ability to respond to patient emotions after medical harm.” After assessment completion, residents were asked to rate the VCA app’s ease of use and the relevance of the cases. They were also asked to rate their performance in the domains of accountability, clear explanation, apology, empathy, caring, and overall response (Multimedia Appendix 2). The items were rated on a 5-point Likert scale that was labeled with “Poor,” “Fair,” “Good,” “Very good,” and “Excellent.” Before time 2, residents were asked if they had “incorporated the feedback into how [they] communicate with patients generally (not just communication about medical harm events).” Due to a technical error, this second survey was not shown to OBGYN residents.

### Analysis

We sought at least 8 raters per response after removing raters with indications of inattention or low contributions to reliability [28]. Ratings were aggregated across raters, items, and vignettes to create an overall score for each resident at time 1 and at time 2. Scores were created by averaging multiple responses to 6 Likert scale questions that were designed to assess general communication skills; thus, the continuous scores presented in this paper were derived from ordinal approximations of continuous variables [29,30]. The reliability and generalizability of the representative cases were analyzed and reported separately [23]. We used a 2×3 split-plot ANOVA to test the effects of time (pre-post) and specialty on communication ratings.

We used paired samples 2-tailed *t* tests to examine whether residents’ self-confidence in their ability to share information and respond to emotions increased from time 1 to time 2. To determine if self-confidence was related to actual ratings, data were subjected to multiple linear regression analyses wherein residents’ self-confidence in their abilities predicted pre- and postfeedback ratings. To determine if changes in such confidence from time 1 to time 2 were associated with changes in actual ratings, difference scores were created; one score represented the difference in residents’ ratings between rounds, and the other represented the difference in residents’ reported self-confidence in their abilities between rounds. Difference scores were subjected to a Pearson correlation analysis. To determine if self-reports of experiences with personally disclosing a harmful error to a patient (“yes” vs “no”) before time 1 were associated with time 1 performance, we used an independent samples 2-tailed *t* test. To determine if years in training were associated with ratings, we performed a Pearson correlation analysis. To determine if physicians’ specialty was associated with prior disclosure experience, we used a chi-square test.

To determine if communication ratings varied with the emotional affects of patients, we created 2 affect scores for each resident. One score represented a resident’s average rating across all vignettes for the two angry affect cases, and the other represented a resident’s average rating for the two sad affect cases. Scores were subjected to a paired samples 2-tailed *t* test.

## Results

### Demographics and User Experience

Of the 238 residents from all specialty departments who were contacted to volunteer for this study, 62 (26%) completed the first two VCA cases (time 1) and received feedback (Table 1). Of these 62 residents, 48 (77%) completed the postfeedback cases (time 2). Less than half of all residents (27/62, 44%) reported prior experience with disclosing medical harm to patients; experience differed significantly among specialties (*P*<.001). Further, 1 of 17 (6%) pathology residents reported previously participating in such conversations, whereas 15 of 23 (65%) OBGYN residents and 11 of 22 (50%) internal medicine residents reported prior experience with such conversations.

**Table 1:** Characteristics and study completion of the 62 resident physicians who participated in this pre-post study of crowdsourced Video-based Communication Assessment (VCA) app ratings.

Characteristics			Specialty, n (%)				
			Obstetrics and gynecology (n=23)		Internal medicine (n=22)		Pathology (n=17)
Sex (female)			19 (83)		15 (68)		9 (53)
**Postgraduate year**							
	1	6 (26)		10 (46)		5 (29)	
	2	6 (26)		6 (27)		4 (24)	
	3	5 (22)		6 (27)		3 (18)	
	4	6 (26)		0 (0)		5 (29)	
Completed the VCA at time 2			15 (65)		17 (77)		16 (94)

Prior to time 2, of the 48 returning residents, 23 completed a survey about incorporating the VCA feedback in general communications with patients. About half (11/23, 48%) agreed or strongly agreed that they had incorporated the feedback, 39.1% (9/23) neither agreed nor disagreed, and 13% (3/23) disagreed that they had incorporated the feedback.

Of the 62 residents, 38 (61%) completed surveys about the VCA app. A majority (32/38, 84%) found the cases to be “very much” or “completely” relevant to their practice. Additionally, 71% (27/38) found the VCA app to be “very much” or “completely” easy to navigate. We achieved a mean of 8.63 crowdsourced raters per case after removing raters with low contributions to reliability, resulting in an average cost of US $8.90 to rate the responses from 1 resident.

### Changes in Resident Communication Ratings From Time 1 to Time 2

Based on the ANOVA for examining changes in mean resident communication ratings from time 1 to time 2, we found that residents were rated significantly higher at time 2 (mean 3.75, SD 0.16) than at time 1 (mean 3.53, SD 0.25; *P*<.001).

### Self-confidence and Communication Ratings

Among the 30 residents who completed surveys at both time points, confidence in the ability to share information about medical harm increased from time 1 (mean 2.87, SD 0.73) to time 2 (mean 3.47, SD 0.63; *P*<.001). Residents’ confidence in their ability to respond to patients’ and families’ emotions after medical harm events also increased from time 1 (mean 3.20, SD 0.71) to time 2 (mean 3.60, SD 0.72; *P*=.005). The multiple linear regression analysis showed no relationship between confidence in such abilities and performance on either the prefeedback ratings or postfeedback ratings. Based on the difference scores evaluated with the Pearson correlation analysis, we found no relationship between increases in confidence over time and increases in ratings over time.

### Self-reported Disclosure Experience, Specialty, and Years in Training

No differences in communication ratings based on specialty were detected. We found no significant relationship between residents’ years in training and overall communication ratings (*P*=.44). However, residents who reported prior experience with disclosing medical harm to patients performed better at time 1 (mean 3.63, SD 0.23) compared to those without prior disclosure experience (mean 3.46, SD 0.25; *P*=.02).

### Ratings by Patient Affect

Residents’ communication was rated significantly higher for angry cases (mean 3.69, SD 0.22) versus sad cases (mean 3.58, SD 0.21; *P*=.01).

### Self-reported Performance in Error Disclosure Domains

After their first VCA use, residents’ mean self-rating of their overall response was 3.82 (SD 0.80). Residents reported their performance in sincerely expressing regret to patients (mean 4.05, SD 0.77), acknowledging and validating patients’ emotions (mean 4.00, SD 0.66), showing that they care about the patient (mean 3.79, SD 0.70), expressing accountability for the harmful event (mean 3.68, SD 0.70), and explaining things in a way that the patient could understand (mean 3.45, SD 0.80).

## Discussion

### Principal Findings

We measured laypeople’s assessment of residents’ error disclosure skills before and after they received numerical and written feedback. Residents’ mean ratings on a 5-point scale improved from 3.53 at baseline to 3.75 after feedback (*P*<.001). This finding provides novel evidence that simulated practice and feedback from laypeople can improve resident physicians’ error disclosure communication. The VCA app represents a novel, scalable, and statistically reliable tool for educators seeking to satisfy the ACGME mandate that residents “participate in the disclosure of patient safety events, real or simulated” [5]. Our findings indicate that the VCA app can be used across multiple specialties. This tool could particularly support educators and residents in diagnostic specialties, such as pathology, who may have fewer opportunities to participate in real-life error disclosure.

Consistent with prior literature about the accuracy of physician self-assessment [24,31], we found that residents’ confidence in their error disclosure skills did not correlate with laypeople’s ratings for these skills. Although physicians should still reflect on their performance, educators can emphasize the use of crowdsourced ratings as a more patient-centered and reliable way to assess error disclosure preparedness. This finding aligns with recommendations by disclosure experts that physicians should seek advice before discussing harmful events with patients [32,33]. Coaches often use brief practice and feedback to help clinicians recognize ineffective communication habits and phrasing before actual disclosures.

The higher ratings of residents when addressing patients with an angry affect versus patients with a sad affect warrant further study. This finding runs counter to predictions that residents might react defensively to confrontational, angry patients, which would be expected to result in lower communication ratings. One possible explanation for this finding is that angry patients’ challenging comments precipitated the explicit acknowledgement of their anger, whereas sad, withdrawn patients did not prompt a direct expression of acknowledgment or support. This would exacerbate existing physician tendencies to avoid discussing patient emotions, as indicated by observations that only 55% of attending surgeons who perform simulated error disclosure attempt to acknowledge or validate patients’ emotions [34]. A second hypothesis is that residents feel less shame when causing anger rather than sadness and respond readily with supportive expressions. Alternatively, the residents in this study may have been exposed to different instructions (ie, outside of this study) for handling patient emotions. Future research could pair an analysis of VCA response content with novel surveys about raters’ expectations for emotional support to characterize effective approaches for specific patient emotions. Error disclosure curricula should prepare trainees to tailor their communication approach to different patient and family emotional responses [35].

### Strengths and Limitations

Our work has limitations. First, the VCA app assesses the skills needed for effective communication after harm but excludes other important areas, such as nonverbal communication. This limitation is offset by the strength that participants received actionable and focused feedback about their phrasing, which addresses a top concern of physicians and helps with not overwhelming them with advice across multiple domains [1,3]. Second, residents did not receive just-in-time coaching or a lecture on error disclosure—practices that might improve performance. Third, this study did not assess long-term skill maintenance. Fourth, the educational significance of the effect size is unknown, as we did not establish a threshold for either competence or mastery. Fifth, the limitations of the study population include recruitment at a single academic center and heterogeneity in the cohort’s training levels. A minority of eligible internal medicine residents participated (22/183, 12%), and not all participants completed all cases, which may have introduced selection bias. To address this, subsequent studies should be embedded in mandatory curricula rather than be based on volunteer participation. Sixth, we did not collect data that would allow us to explain why some residents reported that they did not incorporate the feedback into their communication practices or why some did not complete the cases at the second time point; these findings warrant further study. The efficacy and real-world implementation of this work remain unknown; we tested the VCA app as a stand-alone intervention instead of incorporating it into a longitudinal curriculum with a lecture. Lastly, we did not simultaneously measure faculty ratings of residents. Although this limited our ability to make comparisons between faculty and crowdsourced laypeople, we do not believe that this is a weakness. Rather, it highlights 2 key strengths of the VCA app. First, laypeople directly represent the ultimate arbiters of physician communication effectiveness—patients themselves. Second, crowdsourced laypeople can be recruited rapidly for statistically reliable sample sizes and at lower costs when compared to faculty.

### Conclusion

The VCA app for error disclosure allows users to practice in a safe environment, provides formative feedback, and appears to facilitate skill acquisition. If these findings are replicated, the VCA app will likely offer a scalable way for residency leaders to meet ACGME mandates and assess residents’ skills. Yet, important questions remain about how best to incorporate the VCA app in graduate medical education. Multisite confirmatory studies should examine the effectiveness of using the VCA app in conjunction with didactic sessions on error disclosure and coaching from teaching faculty, as well as longitudinal skill development. Additional work could determine the efficacy of the VCA app for other challenging communication scenarios and for other learner groups, including practicing physicians. Finally, the viewpoints of residency leaders and residents about VCA acceptability, feasibility, and appropriateness will be needed to ensure adoption and sustainable use.

## Appendix

Multimedia Appendix 1 Text of the 12 cases used for the error disclosure video communication assessment.

Multimedia Appendix 2 Survey instruments used in a trial of crowdsourced ratings and feedback about resident physicians' adverse event communication skills.

Multimedia Appendix 3 Deidentified data of average ratings from crowdsourced laypeople for residents’ error disclosure by patient affect (angry vs sad).

Multimedia Appendix 4 Deidentified data of survey responses from residents who used the Video-based Communication Assessment app for error disclosure practice.

## Abbreviations

ACGME: Accreditation Council for Graduate Medical Education
MTurk: Mechanical Turk
OBGYN: obstetrics and gynecology
VCA: Video-based Communication Assessment

## Data Availability

All data generated or analyzed during this study are included in this published article and its supplementary information files (Multimedia Appendices 3 and 4).

## References

[1] Gallagher TH, Garbutt JM, Waterman AD, Flum DR, Larson EB, Waterman BM, Dunagan WC, Fraser VJ, Levinson W (2006). Choosing your words carefully: how physicians would disclose harmful medical errors to patients. Arch Intern Med.

[2] Delbanco T, Bell SK (2007). Guilty, afraid, and alone--struggling with medical error. N Engl J Med.

[3] Gallagher TH, Waterman AD, Garbutt JM, Kapp JM, Chan DK, Dunagan WC, Fraser VJ, Levinson W (2006). US and Canadian physicians' attitudes and experiences regarding disclosing errors to patients. Arch Intern Med.

[4] White AA, Bell SK, Krauss MJ, Garbutt J, Dunagan WC, Fraser VJ, Levinson W, Larson EB, Gallagher TH (2011). How trainees would disclose medical errors: educational implications for training programmes. Med Educ.

[5] (2018). ACGME: Common program requirements (residency). Accreditation Council for Graduate Medical Education.

[6] Koh NJ, Wagner R, Newton RC, Kuhn CM, Co JPT, Weiss KB (2021). CLER: National report of findings 2021. Accreditation Council for Graduate Medical Education.

[7] Stroud L, Wong BM, Hollenberg E, Levinson W (2013). Teaching medical error disclosure to physicians-in-training: a scoping review. Acad Med.

[8] White AA, Gallagher TH, Krauss MJ, Garbutt J, Waterman AD, Dunagan WC, Fraser VJ, Levinson W, Larson EB (2008). The attitudes and experiences of trainees regarding disclosing medical errors to patients. Acad Med.

[9] Gallagher TH (2009). A 62-year-old woman with skin cancer who experienced wrong-site surgery: review of medical error. JAMA.

[10] Stroud L, McIlroy J, Levinson W (2009). Skills of internal medicine residents in disclosing medical errors: a study using standardized patients. Acad Med.

[11] Patrício MF, Julião M, Fareleira F, Carneiro AV (2013). Is the OSCE a feasible tool to assess competencies in undergraduate medical education?. Med Teach.

[12] Adamo G (2003). Simulated and standardized patients in OSCEs: achievements and challenges 1992-2003. Med Teach.

[13] Jacobs AC, van Jaarsveldt DE (2016). 'The character rests heavily within me': drama students as standardized patients in mental health nursing education. J Psychiatr Ment Health Nurs.

[14] McDonough KA, White AA, Odegard PS, Shannon SE (2017). Interprofessional error disclosure training for medical, nursing, pharmacy, dental, and physician assistant students. MedEdPORTAL.

[15] Martinez W, Browning D, Varrin P, Lee BS, Bell SK (2019). Increasing patient-clinician concordance about medical error disclosure through the patient TIPS model. J Patient Saf.

[16] Mazor KM, King AM, Hoppe RB, Kochersberger AO, Yan J, Reim JD (2019). Video-Based Communication Assessment: Development of an innovative system for assessing clinician-patient communication. JMIR Med Educ.

[17] (2017). Introducing the VCA. VCA NBME YouTube page.

[18] Blanch-Hartigan D, Hall JA, Krupat E, Irish JT (2013). Can naive viewers put themselves in the patients' shoes?: reliability and validity of the analogue patient methodology. Med Care.

[19] Sheehan KB (2017). Crowdsourcing research: Data collection with Amazon’s Mechanical Turk. Commun Monogr.

[20] Buhrmester M, Kwang T, Gosling SD (2011). Amazon's Mechanical Turk: A new source of inexpensive, yet high-quality, data?. Perspect Psychol Sci.

[21] Mortensen K, Hughes TL (2018). Comparing Amazon's Mechanical Turk platform to conventional data collection methods in the health and medical research literature. J Gen Intern Med.

[22] Mazor KM, King AM, Hoppe RB, D'Addario A, Musselman TG, Tallia AF, Gallagher TH (2021). Using crowdsourced analog patients to provide feedback on physician communication skills. Patient Educ Couns.

[23] White AA, King AM, D'Addario AE, Brigham KB, Dintzis S, Fay EE, Gallagher TH, Mazor KM (2022). Video-based communication assessment of physician error disclosure skills by crowdsourced laypeople and patient advocates who experienced medical harm: Reliability assessment with generalizability theory. JMIR Med Educ.

[24] Davis DA, Mazmanian PE, Fordis M, Van Harrison R, Thorpe KE, Perrier L (2006). Accuracy of physician self-assessment compared with observed measures of competence: a systematic review. JAMA.

[25] Diaczok BJ, Brennan S, Levine D, Hilu R, Thati N, Kruer J, Ahsan S, McNally P, Pieper D (2020). Comparison of resident self-evaluation to standardized patient evaluators in a multi-institutional objective structured clinical examination: Objectively measuring residents' communication and counseling skills. Simul Healthc.

[26] GUIDANCE exempt research. UW Research.

[27] (2020). Internal medicine milestones. Accreditation Council for Graduate Medical Education.

[28] Vriesema CC, Gehlbach H (2021). Assessing survey satisficing: The impact of unmotivated questionnaire responding on data quality. Educ Res.

[29] Norman G (2010). Non-cognitive factors in health sciences education: from the clinic floor to the cutting room floor. Adv Health Sci Educ Theory Pract.

[30] Sullivan GM, Artino AR Jr (2013). Analyzing and interpreting data from likert-type scales. J Grad Med Educ.

[31] Gude T, Finset A, Anvik T, Bærheim A, Fasmer OB, Grimstad H, Vaglum P (2017). Do medical students and young physicians assess reliably their self-efficacy regarding communication skills? A prospective study from end of medical school until end of internship. BMC Med Educ.

[32] White AA, Brock DM, McCotter PI, Shannon SE, Gallagher TH (2017). Implementing an error disclosure coaching model: A multicenter case study. J Healthc Risk Manag.

[33] Shapiro J, Robins L, Galowitz P, Gallagher TH, Bell S (2021). Disclosure coaching: An ask-tell-ask model to support clinicians in disclosure conversations. J Patient Saf.

[34] Chan DK, Gallagher TH, Reznick R, Levinson W (2005). How surgeons disclose medical errors to patients: a study using standardized patients. Surgery.

[35] Bialer PA, Kissane D, Brown R, Levin T, Bylund C (2011). Responding to patient anger: development and evaluation of an oncology communication skills training module. Palliat Support Care.

