# Supplementary material for: Effects of Practicing With and Obtaining Crowdsourced Feedback From the Video-Based Communication Assessment App on Resident Physicians’ Adverse Event Communication Skills: Pre-post Trial
Source: JMIR Med Educ. 2022 Oct 3;8(4):e40758. doi: 10.2196/40758 (PMC9577713; doi:10.2196/40758)
Supplement: Multimedia Appendix 1 [file mededu_v8i3e40758_app1.docx]

**Appendix 1**

| **Internal Medicine Cases** | | | | | |
| --- | --- | --- | --- | --- | --- |
| Vignette | | Situation description (to physician) | Situation Description (to rater) | What the patient says | Patient affect |
| 1A | | You are a primary care doctor for a 48-year-old woman with diabetes. Today she mentions a breast lump that is new to her. You review her chart and see a mammogram report from last year had a suspicious calcification with recommendation for biopsy. At her last health maintenance visit you did not document a plan for the result and do not recall seeing it before now. You examine her and the site of the lump corresponds to the location on x-ray. She says: | Lorna Smith visits her primary care doctor to evaluate a new breast lump. She figures it isn't anything serious because she had a mammogram last year and never heard about any abnormal results. The doctor examined her and she changed back to regular clothes. She wants to discuss the lump now and says: | “When I didn’t hear from your office about the mammogram, I assumed everything was normal. Was there any sign of this lump on the test last year?” | Sad |
| 1B | | You've told the patient that there were early warning signs of possible breast cancer on her mammogram one year ago. She says: | Ms. Smith has learned that her mammogram last year showed early signs of possible breast cancer, but nothing was done about it. She is feeling panicked and says: | “This is terrible! I’ve never been more frightened…plus you’re telling me that we might have known about it a long time ago!” |  |
| 1C | | You've acknowledged how upsetting the error is. The patient now understands that there were early warning signs of possible breast cancer on her mammogram. She says: | Ms. Smith feels like the clinic and her doctor have failed her. She asks: | “How could this happen to me? I feel like I can’t trust anyone anymore. How am I supposed to believe your advice in the future?” |  |
| 2A | | A 45-year-old male is admitted to orthopedics ten days ago for a left hip fracture. He was transferred to you with postoperative hyponatremia from SIADH and mental status changes. Postoperative left leg swelling was noted and a duplex scan found a deep venous thrombosis. The patient was started on enoxaparin at the standard dose of 1 mg/kg every 12 hours with daily weights. Four days later, the patient has a massive upper GI bleed requiring six units of blood. An endoscopy finds diffuse gastritis. On reviewing the chart, you notice that the patient’s admission weight is listed as 160 kg, which seems high for the size of the patient. No weights had been recorded since. You ask for the patient to be re-weighed, and his weight is 85 kg, not 160 kg. Based on this faulty admission weight, the patient has been receiving nearly twice as much enoxaparin as he needed, which has led to this GI bleed. The nurse has told him his blood was too thin and he’s asked you to explain further. You are now meeting to disclose the error. You enter the room and he asks: | Dan Iglesias was admitted to the hospital ten days ago for a hip fracture and developed a blood clot in his leg. He was placed on a blood thinner, but developed serious bleeding. The nurse told him his blood was too thin, and that the doctor needed to explain more. The doctor sat down to discuss the situation and the patient says: | “I heard I bled because my blood was too thin, but I haven’t had that problem before. Can you explain what is going on?” | Angry |
| 2B | | You've explained to the patient that he suffered bleeding because an error was made in measuring his weight. However, he remains unclear on who is responsible. As you look at the patient, it is visibly obvious he does not weigh 160 Kg. He asks: | Mr. Iglesias understands that the wrong weight was in his medical chart and it caused him to get too much blood thinner. He asks: | “So you mean all of this bleeding could have been avoided? …whose fault is this?” |  |
| 2C | | You’ve explained that several members of the care team misjudged his weight and none, including yourself, questioned the details in the chart. He asks: | Mr. Iglesias understands that several members of his care team missed chances to recognize and fix the wrong weight in the chart. He asks: | “Do you have any idea how terrible this has been for me?” |  |
| 2D | | You have expressed empathy for his unnecessary suffering. He is coming to terms with the error, but has further questions. He asks: | The doctor has shared information about the mistake, but Dan asks: | “I’m glad you’re leveling with me about what happened, but how am I supposed to trust you now?” |  |
| 3A | | A 65-year-old male with diabetes is admitted for a COPD exacerbation. During medication documentation a pharmacy technician mistakenly enters his insulin as 100 units with meals instead of 10 units, his home dose. When entering computerized admission orders, you do not catch this discrepancy. The nurse does not discuss the dose with the patient and administers it as ordered. The patient develops life-threatening hypoglycemia that is recognized and treated before injury occurs. He requires overnight monitoring in the ICU. His nurse explains he had an insulin overdose, but defers further explanation to you. As you enter the room he says: | Jeff Thompson is admitted for breathing problems and seems to be improving when he is rushed to the intensive care unit for confusion. His nurse tells him that he got too much insulin, a medicine he normally takes at home at a dose of 10 units. The doctor visits to explain the situation. Mr. Thompson says: | “I can’t remember anything from last night, but heard blacked out from low blood sugar. The nurse said I received too much insulin, and that my morning sugar was normal, but wouldn’t say more. I need to know - how much did I get and why was my dose changed?” | Angry |
| 3B | | You've explained that he received a ten-fold overdose. As someone experienced with managing his own diabetes, he understands this was a major and dangerous mistake. He says: | Mr. Thompson now appreciates that he received ten times his usual insulin dose, which causes confusion and could be deadly. He reacts by saying: | Well that’s just great! Do you guys have any idea what you’re doing here?! You nearly killed me!” |  |
| 3C | | You’ve expressed regret for the insulin ordering error and recognized its serious nature. He continues to want a deeper understanding of the situation and asks further questions. | Jeff wants more information about what the hospital will do next. He asks: | “When I worked in manufacturing, you can bet I’d lose my job for something like this. Is someone going to investigate you and the nurse? How do I know you won’t do this to another patient?” |  |
| 3D | | You've explained how the hospital will perform a root cause analysis and determine ways to prevent recurrence. He says: | Mr. Thompson realizes that intensive care is very expensive and asks: | “I’m not happy about this, but I’m glad no permanent harm occurred. However, I need to know – who’s going to pay for all this extra intensive care?” |  |
| 4A | | You are a primary care doctor for a 65-year-old woman with COPD. She came to your clinic last week with dyspnea and inspiratory chest pain. After a history, physical, and exam, you thought a COPD exacerbation was most likely. You treated her with nebulizers in the office and she felt significantly better, although remained tachycardic. Hoping to save her a lengthy visit to the ER, you obtained an EKG and chest x-ray with reassuring results and prescribed outpatient steroids and increased bronchodilators. Two days later an intensivist in your healthcare system calls to inform you the patient collapsed at home and was diagnosed with a massive PE. She underwent thrombolysis and appears to be making a promising recovery. She now knows her symptoms in the office were from the PE and wants to discuss this with you. You enter the room and she says: | Carol Walker saw her doctor last week for chest pain and trouble breathing. She has emphysema (also called COPD). Her doctor did an xray and decided to treat her in the office for COPD, rather than send her to the ER for more tests. She felt better after inhalers and went home with a new prescription. Later that day, she collapsed at home and was rushed to the hospital. The intensive care doctors found a dangerous blood clot in her lungs, called a pulmonary embolus (PE), which caused her symptoms. Her primary doctor visits her in the hospital and Carol says: | “I’m really scared. I can’t believe I almost died. I just can’t get over how serious this was for me.” | Sad |
| 4B | | You've told the patient that you reached a premature conclusion about her diagnosis and missed the PE. The patient has further questions about the misdiagnosis. She says: | Ms. Walker understands that her doctor made the wrong diangosis, but is still unsure how it happened or what it means for her health. She asks: | “They put me on blood thinners here, but also had to give a clot-buster medicine that was pretty frightening. And they’ve said I could have long-term breathing problems. Do you think this could have been prevented with an earlier diagnosis?” |  |
| 4C | | You have told the patient that an earlier diagnosis might have prevented the frightening collapse at home and acknowledged that you can't yet predict the long-term health consequences. The patient has further questions about her care. She says: | Ms. Walker is trying to come to terms with her misdiagnosis and tells the doctor: | “This is so terrible. I don’t know who I can trust anymore and don’t really want to ever have to go to the doctor again. I mean, I like you, but I just don’t know how I can go on after something so awful.” |  |
| **OB/GYN Cases** | | | | | |
| Vignette | Situation description (to physician) | | Situation Description (to rater) | What the patient says | Patient affect |
| 1A | A 31-year-old G4P0040 and a history of recurrent pregnancy loss returns to your office for follow-up for a presumed ectopic pregnancy given slowly rising BHCG and no visualized intrauterine pregnancy on initial ultrasound. You recently prescribed two doses of methotrexate. However, on repeat imaging today an intrauterine pregnancy was diagnosed. The ultrasonographer told her that she had an intrauterine pregnancy; the patient is now expecting to see you. You sit down and she says: | | Eva Jha is trying to start a family, but experienced 4 miscarriages. She was recently diagnosed with an ectopic pregnancy (a pregnancy stuck in the fallopian tube rather than the uterus). This is dangerous to the mother so she was prescribed the first of two doses of a medication called methotrexate to abort the pregnancy. However, on a followup ultrasound, the technician sees evidence of a pregnancy in the uterus. The doctor comes to discuss the news and she says: | “I want you to explain to me exactly what is going on. You told me I had an ectopic pregnancy, and that I needed that medication, but on my ultrasound today I saw a heartbeat….” | Angry |
| 1B | You have explained that you misdiagnosed the ectopic pregnancy, and because of that the methotrexate was recommended. You have explained that the medication is teratogenic and poses significant risks to her developing pregnancy. She says: | | Ms. Jha understands there was a misdiagnosis that led to the methotrexate prescription. She understands that her pregnancy may not progress now because of the dose of methotrexate. She says: | “I can’t believe you did this to me. It’s been so hard for me to get pregnant, and this was my chance, and now I might have hurt my baby with the medication you recommended?” |  |
| 1C | You have acknowledged how terrible and upsetting this mistake has been. She asks: | | The doctor has acknowledged how terrible and upsetting this mistake has been. She asks: | “How can I possibly trust you again as my doctor?” |  |
| 1D | You have expressed understanding that her trust in you has been damaged. She asks: | | The doctor has expressed understanding that her trust in you has been damaged. She asks: | “Can you even begin to understand what I’ve been through? I’ve lost so many babies already.” |  |
| 2A | A 48-year-old G2P2 returns to your office for follow-up after an open total abdominal hysterectomy for large fibroids. She initially recovered well after the surgery, but then was readmitted after she developed fevers and abdominal pain, and on CT abdomen/pelvis was found to have a retained lap sponge. The ER doctor has told her that she has a retained object and asked you to come explain the plan. You now need to perform an ex-lap to remove the sponge. You meet with her and she asks: | | Because of painful fibroids, Jessica Studor had her uterus removed through a large open abdominal surgery. She initially recovered well, but had to return to the hospital with fevers and pain. A scan was done and the ER doctor told her a sponge was left inside her abdomen. She asks to speak with the gynecologist who did the operation. She asks: | “I just learned you left something inside my body. I want you to explain to me exactly what happened.” | Angry |
| 2B | You have explained that during her surgery a lap sponge was accidentally left inside of her, which led to her infection and requirement of a second surgery. You have explained this happened despite efforts to count the sponges used in the case. She says: | | She understands that a surgical sponge was supposed to be removed, but was forgotten, despite a usual practice of counting sponges at the end of surgery. It is now causing an infection and another surgery is required to retreive it. She says: | Well that’s just great! Do you guys have any idea what you’re doing here?! You nearly killed me!” |  |
| 2C | You have acknowledged that this was a serious and harmful error. She says: | | The doctor has acknowledged that this was a serious and harmful error. She says: | “In my line of work, you can bet I’d lose my job over a mistake like this. Is someone going to investigate this? How do I know you won’t do this to another patient?” |  |
| 2D | You have explained that the hospital will conduct an analysis to understand how the mistake happened and improve systems to prevent it from happening to another patient. She asks: | | The doctor explained that the hospital will conduct an analysis to understand how the mistake happened and improve systems to prevent it from happening to another patient. She asks: | “Do you even understand how hard this has been for me?” |  |
| 3A | A 27-year-old G2P2 is 2 days postpartum following an emergency c-section. She presented with PROM, and underwent augmentation with Pitocin. She reached 7cm and was noted to have two late decelerations. The plan was to turn the Pitocin off, and do the usual resuscitative measures, including a bolus of lactated ringers. The nurse accidentally gave her a 500cc bolus of Pitocin, mistaking it for the LR. She then had a tetanic contraction, terminal bradycardia, and underwent an emergency c-section. The baby went briefly to the NICU for monitoring and was ready for discharge home on postpartum day #2.  During the emergency, the patient noticed that something had gone wrong and is now asking to meet with you. You sit down and she says: | | Susie Beltran had her second baby two days ago. During labor she had prolonged abnormal contractions that seemed to hurt the baby, leading the doctor to do an emergency c-section. She had a sense that something had gone wrong by the way the nurses and doctors had talked with each other. The baby went to the neonatal intesive care unit, but is doing well now. She asks to talk to her doctor and says: | “During the craziness before my c-section, I could tell that something had gone wrong. However, I was scared and in too much pain to ask until now. I’m ready now – I want you to tell me exactly what happened. Why did I have an emergency c-section?” | Sad |
| 3B | You have explained that the emergency c-section was performed due to fetal distress. You explained that it was an error that she received a bolus of Pitocin instead of a fluid bolus, and this likely triggered the emergency. She says: | | Ms. Beltran understands that an error occurred in which she received too much Pitocin, a medication to encourage labor. A c-section was necessary to save the baby after the overdose. She says to her doctor: | “I can’t believe you let this happen…you risked my baby’s life, and my own. I never wanted a c-section and it happened anyway!” |  |
| 3C | You have acknowledged how upsetting this error has been and expressed regret for the unwanted c-section. She says: | | The doctor acknowledged how upsetting this error has been and expressed regret for the unwanted c-section. She says: | “I don’t see how I’ll ever be able to trust you again as my doctor, or this hospital.” |  |
| 3D | You expressed understanding that her trust in you and in the hospital has been damaged. She asks: | | The doctor expressed understanding that Annabelle's trust in the doctor and the hospital has been damaged. She asks: | “Can you even begin to understand what I’ve been through?” |  |
| 4A | Ms. Miller is a 32 yo G2P2002, now 4 weeks postpartum from a repeat c-section in the setting of second stage arrest (failed TOLAC). The fetal head extraction was difficult, and resulted in two very large uterine extensions bilaterally, which were repaired in the usual fashion. No cystoscopy was done at the conclusion of the case. Patient was discharged home on POD#2. She represented 7 days later with increasing abdominal pain and distention and was ultimately diagnosed with a ureteral injury requiring stenting. She learned from a nurse at the time of stenting that stents are only required “when something goes wrong”. She has asked to speak with you about her care. You sit down and she says: | | Lisa Miller had c-section 4 weeks ago to deliver her second child. A week later she returned to the hospital with pain and swelling in her belly. She was diagnosed with blockage of the ureter, the tube connecting the kidney to the bladder. A stent was placed to open the blockage. A nurse told her "stents are only needed when something goes wrong". She made a follow up appointment to speak with her doctor and says: | “Doctor - I need more information about my care, because I believe the problems with my blocked kidneys weren’t supposed to happen. However, nobody has given me a good explanation. I need you to tell me what happened and whether it was a mistake…” | Sad |
| 4B | You have explained that her C-Section was difficult, and resulted in two large tears in her uterus. Further, an injury to her ureter was not an expected part of the operation, and you missed the diagnosis at the time. She says: | | Ms. Miller now understands that the c-section was difficult and caused a large tear of her uterus. An injury must have occurred to the nearby ureter and was not diagnosed at the time. She says: | “I can’t believe this happened to me – it is so terrible! How could you have missed an injury to my ureter?” |  |
| 4C | You have explained that a cystoscopy was not performed, but could have caught the problem earlier. You express regret that you did not check for this. She says: | | She understands that a camera could have been put in her bladder to check for this injury, but this was not done. She says: | “I don’t see how I’ll ever be able to trust doctors again after this…even just coming to your office today made me really anxious.” |  |
| 4D | You have acknowledged how upsetting this has been and that her trust in you is damaged. She asks: | | The doctor has acknowledged how this has damaged Lisa's trust in her care team. She asks: | “How will I afford the bills from all of this extra care? I’m in debt and already on the edge of keeping things together. Who is responsible for all of this?” |  |
| **Pathology Cases** | | | | | |
| Vignette | Situation description (to physician) | | Situation Description (to rater) | What the patient says | Patient affect |
| 1A | A 57-year-old female underwent a breast needle biopsy that demonstrated grade 3 invasive ductal carcinoma. The immunohistochemical studies are interpreted as follows: • Negative for estrogen and progesterone receptor expression • Positive for HER2 protein over-expression The patient is treated with chemotherapy optimized for HER2 positive carcinoma for 6 months.  The patient transfers to another hospital and the reviewing pathologist questions the previous interpretation of the HER2 stain. Subsequent fluorescence in situ hybridization studies (HER2 FISH) confirm that the carcinoma is not amplified for the HER2 gene and is negative for HER2 over-expression. The patient has not been receiving the optimum therapy for her locally advanced invasive ductal carcinoma of the breast, but it is unclear how this will affect her prognosis. Her oncologist has told her that a diagnostic error in pathology has led to treatment with extra chemotherapy that was unnecessary. She asked to speak with you about the error. You sit down and she says: | | Lucia Rossi was diagnosed with breast cancer. She received 6 months of a specific chemotherapy based on tests on her biopsy for high levels of a gene called "HER2". She transferred care to another hospital and their pathologist reviewed her biopsy. The new pathologist found that her cancer did not have high levels of HER2. Her oncologist tells her that a diagnostic error has led to treatment with unnecessary chemotherapy. She meets with the pathologist involved and says: | “You’re the doctor who originally read my biopsy, right? It’s my understanding that I’ve been receiving the wrong kind of chemo. Please explain how this happened.” | Angry |
| 1B | You explained that you misjudged the appearance of cells under the microscope, leading to the misdiagnosis. She asks: | | Ms. Rossi understands that the pathologist misjudged the appearance of cells under the microscope, leading to the misdiagnosis. She says: | “I’m really angry about wasting all that time with the wrong treatment – how could you let this happen?” |  |
| 1C | You've explained that you overestimated the degree of HER-2 expression. You expressed regret for the additional treatment she received as a result. She says: | | Ms. Rossi learned that the doctor misjudged the color of the cells, which determines whether a drug for HER2 will work. The doctor expressed regret for the mistake. She asks: | “I really need to be able to have faith in my care team but I don’t know who I can trust anymore!” |  |
| 1D | You've acknowledged how this error damaged her trust in you and the hospital. She says: | | The doctor acknowledged that this error has damaged Ms. Rossi's trust in the hospital and the doctor. She asks: | “Do you have any idea how hard this has been for me? What if I hadn’t changed hospitals and this was never discovered?” |  |
| 2A | A 56-year-old female with an extensive tobacco history, pain, and swelling of the mandible was sent for a buccal mucosal biopsy. You review the slides, see widespread inflammatory change, and diagnose squamous cell carcinoma. The patient undergoes a partial mandibular resection for presumed squamous cell carcinoma eroding into bone. A few days later, the definitive resection specimen reveals chronic osteomyelitis with no evidence of malignancy. The appropriate therapy for this diagnosis would have involved antibiotic therapy and surgical debridement. The treating clinician has explained to the patient that a diagnostic error occurred, and the patient has asked to meet with you for further information. You sit down and she says: | | Stacy Hill is a heavy smoker who developed jaw swelling. A biopsy is taken and the pathologist diagnoses cancer. She has part of her jaw surgically removed. Several days later the surgeon tells her that examination of the removed section of her jaw shows an infection rather than a cancer. She could have been treated with antibiotics and a minor surgery. She asks to meet the pathologist involved and says: | So you’re the doctor that read my biopsy – it sounds like I didn’t get the right diagnosis, but I just wanted to hear straight from you about what happened.” | Sad |
| 2B | You have explained that you misjudged the significance of the inflammation on the biopsy. She says: | | She understands that the pathologist saw a lot of inflammation on the biopsy and misjudged the cause of the inflammation. She says: | "I thought I had cancer and went through major surgery – and now I find out I didn’t need to! I’m so upset with this whole process and don’t know who I can trust anymore!” |  |
| 2C | You have expressed regret for the unnecessary surgery that resulted from this misdiagnosis. She asks: | | The doctor has expressed regret for the unnecessary surgery that occurred after this misdiagnosis. She asks: | “Do you have any idea how hard this has been for me? It has been so hard to eat and I need cosmetic surgery to look normal again…” |  |
| 2D | You have acknowledged that has been painful and upsetting. She asks: | | The doctor has acknowledged that this mistake has been painful and upsetting. She asks: | “I just can’t imagine this happening to anyone else. How can you make sure that nobody else has to suffer like me?” |  |
| 3A | A 43-year-old male patient had a routine renal biopsy 3 years post renal transplantation. The pathology report identified acute rejection, necessitating hospitalization for pulse corticosteroid treatment, which caused Mr. Jones to become hyperglycemic and develop oral candidiasis.  A different patient with clear clinical signs of rejection underwent renal biopsy on the same day. Surprisingly, the biopsy showed no evidence of rejection. The nephrologist contacted the renal pathology team, who discovered the two patients’ biopsies had been mislabeled and switched in specimen processing. After the treating physician informed Mr. Jones of the error, he requested a meeting with you the following week. You enter the room and he asks: | | John Jones had a kidney transplant 3 years ago. A routine annual biopsy detects transplant rejection. He feels fine, but is put on high doses of anti-inflammatory steroids. This causes side effects like a mouth infection and high blood sugar. A week later, his kidney doctor calls to say that his biopsy specimen was switched with another patient's in the lab and he doesn't have transplant rejection after all. He asks to meet with the director of pathology and asks: | “I needed to meet with you because I can’t believe this happened! How did my biopsy end up in the wrong place?” | Angry |
| 3B | You've explained to the patient that there was a human error in labeling, leading to the wrong diagnosis being assigned in his chart. He asks: | | He understands that a human error was made in labeling his biopsy, leading to the wrong diagnosis. He asks: | “So who is to blame for this? What kind of investigation happens to sort this out?” |  |
| 3C | You've explained that the hospital will conduct a thorough investigation to understand all of the factors that caused the error. You've explained that multiple members of the care team share accountability. He says: | | John understands that the hospital will conduct an investigation to determine the causes of the mistake. He asks: | “After everything I’ve been through, how can I trust that my future test results won’t be mixed up?” |  |
| 3D | You have committed to making system changes after the investigation and to sharing the plan with colleagues. He asks: | | John has learned that the doctor will work with the team in specimen processing to reduce the chance of this mistake occurring again. He asks: | “Do you have any understanding of what I’ve been through?” |  |
| 4A | A 52 yo man undergoes surgery to remove a parotid gland nodule. Upon reviewing the slides, you rendered the diagnosis of pleomorphic adenoma, a common benign salivary gland neoplasm. You dictated a report and the transcriptionist mistakenly enters the diagnosis of “pleomorphic adenocarcinoma”. You did not notice the transcription error and finalized the transcribed report with the incorrect diagnosis of pleomorphic adenocarcinoma (high grade, undifferentiated adenocarcinoma.) At his post-operative visit one week later, the surgeon discusses the unexpected diagnosis of high grade cancer with the patient and schedules appointments with an oncologist and radiation oncologist.  He is discussed at head and neck tumor board. Upon review of the pathology at tumor board, the reporting error is discovered. The pathology report is amended to reflect the correct benign diagnosis. The surgeon informs the patient; he then asks to speak to you to understand the error. He says: | | Jorge Gonzalez has a lump removed from a saliva gland near his jaw. His surgeon gets a report of a serious cancer and tells Billy he needs to meet an oncologist promptly for chemotherapy and radiation. A couple of weeks later, the surgeon calls to say that the report had a typo and that his lump was benign. This has been very disruptive and he meets with the pathologist who reviewed the biopsy. He asks: | “So you’re the doctor that read my biopsy – it sounds like the wrong word was used, but I just wanted to hear straight from you about what happened.” | Sad |
| 4B | You've explained that you made the correct diagnosis, but there was a transcription error that changed the name of his condition to something similar-sounding, but very serious. You explained that you did not catch the error when you reviewed the document. He says: | | He understands that the pathologist made the correct diagnosis, but the dictated report was transcribed with a similar-sounding cancer. The pathologist did not catch the mistake when reviewing the report. He says: | “I thought I had life-ending cancer and almost went through major surgery – and now I find out I didn’t need all that stress! I’m sortof relieved, but also so upset with this whole process and don’t know who I can trust anymore!” |  |
| 4C | You have acknowledged that this has damaged his trust in you and the hospital. He asks: | | The doctor acknowledged that Mr. Gonzalez's trust in his care team has been damaged. He asks: | “Do you have any idea how hard this has been for me? I’ve gone into major depression and submitted a letter of resignation at work….I’ve got to rebuild my life now” |  |
| 4D | You have expressed empathy for the personal harm and pain this error has caused. He asks: | | The doctor has acknowledged how disruptive this mistake has been. He asks: | “I just can’t imagine this happening to anyone else. How can you make sure that nobody else has to suffer like me?” |  |
