# Supplementary material for: Effects of Practicing With and Obtaining Crowdsourced Feedback From the Video-Based Communication Assessment App on Resident Physicians’ Adverse Event Communication Skills: Pre-post Trial
Source: JMIR Med Educ. 2022 Oct 3;8(4):e40758. doi: 10.2196/40758 (PMC9577713; doi:10.2196/40758)
Supplement: Multimedia Appendix 2 [file mededu_v8i3e40758_app2.docx]

**Appendix 2: Survey Instruments used in a trial of crowdsourced rating and feedback about resident physician adverse event communication skills**

Item 1: Survey items completed by *crowdsourced laypeople* regarding physician audio responses to the Video-based Communication Assessment (VCA)

“I would feel this provider was accountable for their actions”,

“I would feel this provider was being honest about what happened”,

“I would feel this provider was sincerely sorry for what happened”,

“I would feel this provider understood how I was feeling”,

“I would feel this provider cared about me”,

“Overall this provider’s response was”

The first five items were rated on a 5-point scale anchored to “Not at all”, “A little”, “Somewhat”, “Very much”, “Completely”

The overall response was rated on a 5 point scale anchored to ““Poor”, “Fair”, “Good”, “Very good”, “Excellent”.

Item 2: Survey items completed by *resident physicians* regarding the VCA, completed after their first use of the VCA.

“This exercise was easy to navigate”

“The patient and situation in this exercise were relevant to my practice”

“I acknowledged and validated how the patient was feeling”

“I showed that I cared about the patient”

“I explained things in a way the patient could understand”

“I sincerely expressed regret to the patient”

“I conveyed accountability for the patient’s harmful event”

“Overall, my response was”

The first seven items were rated on a 1 – 5 Likert scale anchored with labels “Not at all”, “A little”, “Somewhat”, “Very much”, “Completely”.

The overall response was rated on a 5-point scale anchored to “Poor”, “Fair”, “Good”, “Very good”, “Excellent”.
